# Deep Transcriptomic Analysis Reveals the Dynamic Developmental Progression during Early Development of Channel Catfish (*Ictalurus punctatus*)

**DOI:** 10.3390/ijms21155535

**Published:** 2020-08-02

**Authors:** Xiaoli Ma, Baofeng Su, Yuan Tian, Nathan J. C. Backenstose, Zhi Ye, Anthony G. Moss, Thuy-Yen Duong, Xu Wang, Rex A. Dunham

**Affiliations:** 1 School of Fisheries, Aquaculture and Aquatic Sciences, Auburn University, Auburn, AL 36849, USA; xzm0017@auburn.edu (X.M.); subfeng@auburn.edu (B.S.); yzt0059@aubuen.edu (Y.T.); njbackenstose13@gmail.com (N.J.C.B.); zhiy0008@uw.edu (Z.Y.); 2 Alabama Agricultural Experiment Station, Auburn, AL 36849, USA; 3 Department of Biological Sciences, University at Buffalo, Buffalo, NY 14260, USA; 4 Department of Biochemistry, University of Washington, Seattle, WA 98195, USA; 5 Department of Biological Science, Auburn University, Auburn, AL 36849, USA; mossant@auburn.edu; 6 College of Aquaculture and Fisheries, Can Tho University, Can Tho City 94000, Vietnam; thuyyen@ctu.edu.vn; 7 Department of Pathobiology, Auburn University, Auburn, AL 36849, USA; 8 HudsonAlpha Institute for Biotechnology, Huntsville, AL 35806, USA

**Keywords:** early development, channel catfish, RNA sequencing, WGCNA

## Abstract

The transition from fertilized egg to larva in fish is accompanied with various biological processes. We selected seven early developmental stages in channel catfish, *Ictalurus punctatus*, for transcriptome analysis, and covered 22,635 genes with 590 million high-quality RNA-sequencing (seq) reads. Differential expression analysis between neighboring developmental timepoints revealed significantly enriched biological categories associated with growth, development and morphogenesis, which was most evident at 2 vs. 5 days post fertilization (dpf) and 5 vs. 6 dpf. A gene co-expression network was constructed using the Weighted Gene Co-expression Network Analysis (WGCNA) approach and four critical modules were identified. Among candidate hub genes, *GDF10*, *FOXA2*, *HCEA* and *SYCE3* were involved in head formation, egg development and the transverse central element of synaptonemal complexes. *CK1*, *OAZ2*, *DARS1* and *UBE2V2* were mainly associated with regulation of cell cycle, growth, brain development, differentiation and proliferation of enterocytes. *IFI44L* and *ZIP10* were critical for the regulation of immune activity and ion transport. Additionally, *TCK1* and *TGFB1* were related to phosphate transport and regulating cell proliferation. All these genes play vital roles in embryogenesis and regulation of early development. These results serve as a rich dataset for functional genomic studies. Our work reveals new insights of the underlying mechanisms in channel catfish early development.

## 1. Introduction

Catfish (order Siluriformes) is one of the most taxonomically diverse orders, which includes over 12% of all teleost species (about 33,000 teleost species) and 6.2% of all vertebrates (64,000 vertebrates) [1]. Channel catfish (*Ictalurus punctatus*) and its hybrid from mating with blue catfish (*I. furcatus*) males, are the most extensively cultured type of fish in the USA, accounting for a farmgate revenue of $185 million in 2015 [2].

There are many studies concerning the study of channel catfish at the level of transcriptome, such as the liver transcriptome analysis to determine the immune-response modulation in channel catfish [3], the testicular differentiation of channel catfish revealed by transcriptome analysis [4], as well as the genes associated with chamber formation of catfish swim bladder [5]. The study of embryogenesis is critical for a comprehensive understanding of the gene expression patterns and underlying biological changes during early embryonic developmental stages of an organism. There are many studies focused on the morphology of channel catfish during early development [6,7,8,9]. Also, there are many studies concerning the early embryonic development in model species using RNA-sequencing (seq), such as mouse (*Mus musculus*), fruit fly (*Drosophila melanogaster*) and zebrafish (*Danio rerio*) [10,11,12,13]. However, the early development in channel catfish has not been studied at the transcriptome level. This represents a critical knowledge gap that stands in the way of improved understanding of differentiation and growth of the channel catfish. Considering the importance of farmed channel catfish to the economy of the southern U.S., there is a pressing need to reveal the developmental processes in as much detail as possible. Channel catfish are one of the most studied catfish, with the first genome assembly released in 2016 [14]. The established genome provides an excellent resource for functional genomic studies and biological research, thereby making the channel catfish an appropriate target for functional studies.

Unlike the genome, the transcriptome is dynamic and should be a good reflection of the cellular function at a specific developmental stage [15]. Accordingly, RNA-Seq analysis should provide gene expression profiles of developing embryos at different stages, as well as the transcriptional structures of genes, which are of great value to the annotation of functional elements in the genome [16]. Weighted gene co-expression network analysis (WGCNA) is a widely used method to correlate modules and concatenate external traits. It can also explore relative timing of genes clustered into the same module, because genes clustered into a module are thought to be highly connective, with potentially have broadly similar functions that contributed to a common goal [17]. WGCNA analysis can further identify hub genes, which are critical for a specific trait or biological process [17]. WGCNA is a powerful method for transcriptome analysis and has been widely applied in studying many different biological contexts, including carcinogenesis, brain imaging and early development in various species [18,19,20].

In this study, we used deep RNA-sequencing to investigate the gene expression profiles of channel catfish during early development stages. Seven early developmental stages, including 2, 5, 6, 7, 8, 9 and 10 days post fertilization (dpf), were selected for transcriptome sequencing and analysis. From the early embryo stage (2 dpf), which sets upon a large yolk, to the adult-like stage (10 dpf), the critical period of channel catfish development and organogenesis was covered. The transcriptome dynamics across early development were provided, which will serve as a blueprint for future investigation of early development and organogenesis. We have identified differentially expressed genes (DEGs) between neighboring developmental stages. The DEGs were used to conduct Gene Ontology (GO) enrichment analysis to reveal the biological functions and determine the co-expressed modules in WGCNA analysis. Eight candidate hub genes, that define eight stage-specific modules, were identified using Cytoscape. The hub genes are expected to be heavily involved in early development in channel catfish.

## 2. Results

### 2.1. Morphology of the Channel Catfish Embryos/Larvae during Early Development

**Figure 1:**
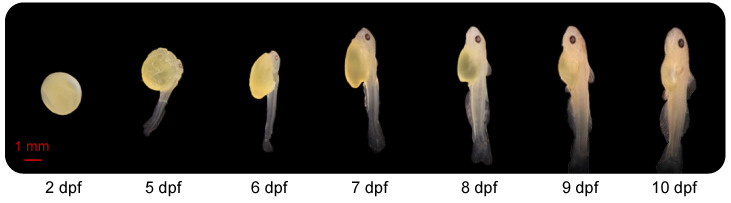
Morphology of the channel catfish, *Ictalurus punctatus*, at different development stages. dpf: days post fertilization.

At 2 dpf, the embryos, developing within the chorion, were approximately 4 mm in length and oval with no eyes (Figure 1). At 5 dpf, the average length was 9 mm, and tail buds were free from the yolk sac. The larval head was close to the yolk sac, eyes were observed, larvae laid on their side in the water and activity of the tail allowed the fish to move slowly. At 6 dpf, the yolk sac was partially absorbed, and the embryo looked more like a larva. The larvae were able to swing their tails slowly, propelling them to the surface of the water. Their average length was 9 mm at this stage. By7 dpf, body length had increased to 11 mm. The dorsal fin had started to develop, and bone could be observed clearly through the translucent body. From days 8 to 10, and especially at 10 dpf, the yolk sac was almost entirely absorbed. Total length was approximately 12–14 mm. The external features, including head, fins, musculature, mouth and barbels, progressively developed toward an adult-like appearance between 8, 9 and 10 dpf.

### 2.2. Global Analysis of Channel Catfish Early Development Transcriptome

**Figure 2:**
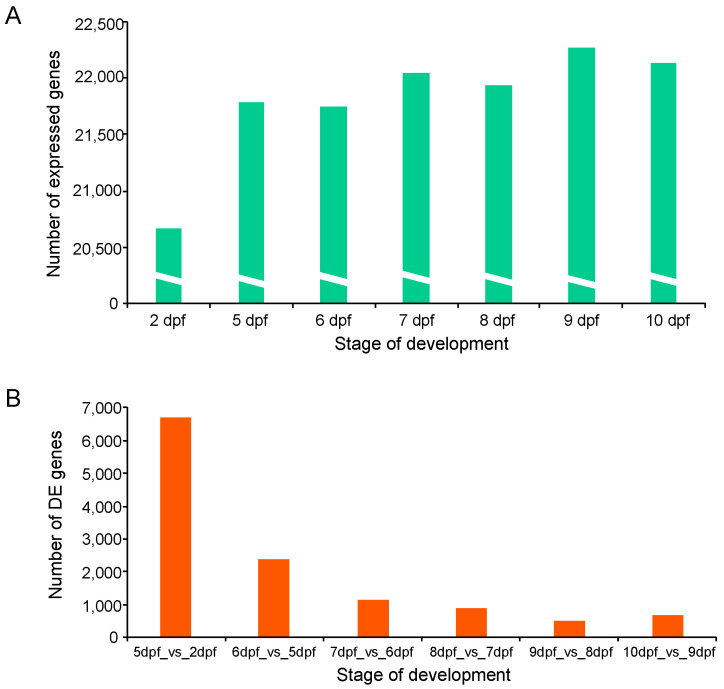
Gene expression during early development in channel catfish, *Ictalurus punctatus*. (**A**) Mean number of expressed genes of two replicates identified at each development stage. (**B**) The number of differentially expressed genes (DEGs) for comparison of each stage with the previous stage.

Transcriptome sequencing resulted in a total of 1259 million raw reads for all samples. After removing low-quality reads with a quality score <25 and reads shorter than 36 bases, more than 591 million high-quality reads were retained for further analysis. Reads from each sample were aligned to the channel catfish reference genome (Supplementary Table S1 and Figure S1). The average number of raw, filtered reads, GC content, number of mapped reads and mapping rate for samples are shown in Supplementary Table S1. From each stage, a total number of 72.26% to 95.06% reads were successfully mapped. Paired-end sequences (2 × 150 bp) were generated with an average trimmed read length ranging from 36 to 135 bp. Gene read counts were normalized using the FPKM (fragments per kilobase of exon model per million reads mapped) method. Genes with a FPKM value smaller than 0.1 were removed. In total, the expression of 22,635 distinct genes was identified. The highest number of expressed genes (22,266) occurred on day 9 post fertilization, while the 2 dpf sample contained the lowest number of expressed genes (20,670) (Figure 2A). A total of 19,415 genes were expressed at all developmental stages (Supplementary Table S2).

### 2.3. Identification of DEGs during Early Development of Channel Catfish

**Figure 3:**
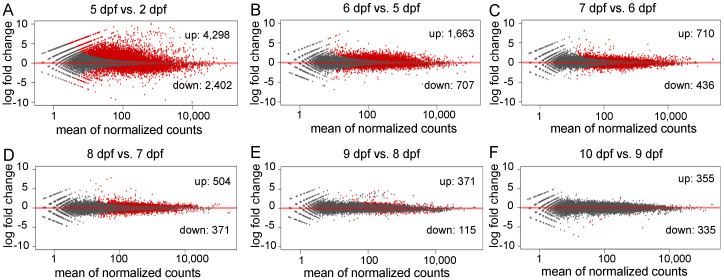
M-A plots (M: log ratio of expression fold change; A: mean of normalized counts) of the differentially expressed genes (DEGs) in different comparisons during early development in channel catfish, *Ictalurus punctatus*. Red dots indicate the downregulation (negative value) or upregulation (positive value). Black dots represent non-DEGs. (**A**) 5 days post fertilization (dpf) vs. 2 dpf, (**B**) 6 vs. 5 dpf, (**C**) 7 vs. 6 dpf, (**D**) 8 vs. 7 dpf, (**E**) 9 vs. 8 dpf and (**F**) 10 vs. 9 dpf.

To investigate genes related to the early development of channel catfish, differential gene expression analysis was conducted among the seven developmental stages in channel catfish using the software package DESeq2. DEGs were identified by comparing two neighboring developmental stages. The number of DEGs varied from 690 (355 upregulated and 335 downregulated) between 10 and 9 dpf, to 6,700 (4298 upregulated and 2402 downregulated) between 5 and 2 dpf (Supplementary Table S3). Overall, the number of DEGs decreased over time during development, except for a slight increase at 10 dpf compared to 9 dpf (Figure 2b). When all of the DEGs at various stages were combined, the majority (91.33%) of the DEGs were detected between the first two pair comparisons (5 vs. 2 dpf and 6 vs. 5 dpf). 7767 of all 8504 DEGs are exclusively present in these two early-stage comparisons, indicating dramatic gene expression changes in the earliest stages of development. These stages are critical for the transitions from fertilized embryos to larvae in channel catfish, which is consistent with the observations of morphological changes. The number and fold-change of DEGs across different developmental stages can be visualized in the M-A (M: log ratio of expression fold change; A: mean of normalized counts) plots (Figure 3).

### 2.4. Gene Ontology Enrichment Analysis of DEGs at Different Stages

**Figure 4:**
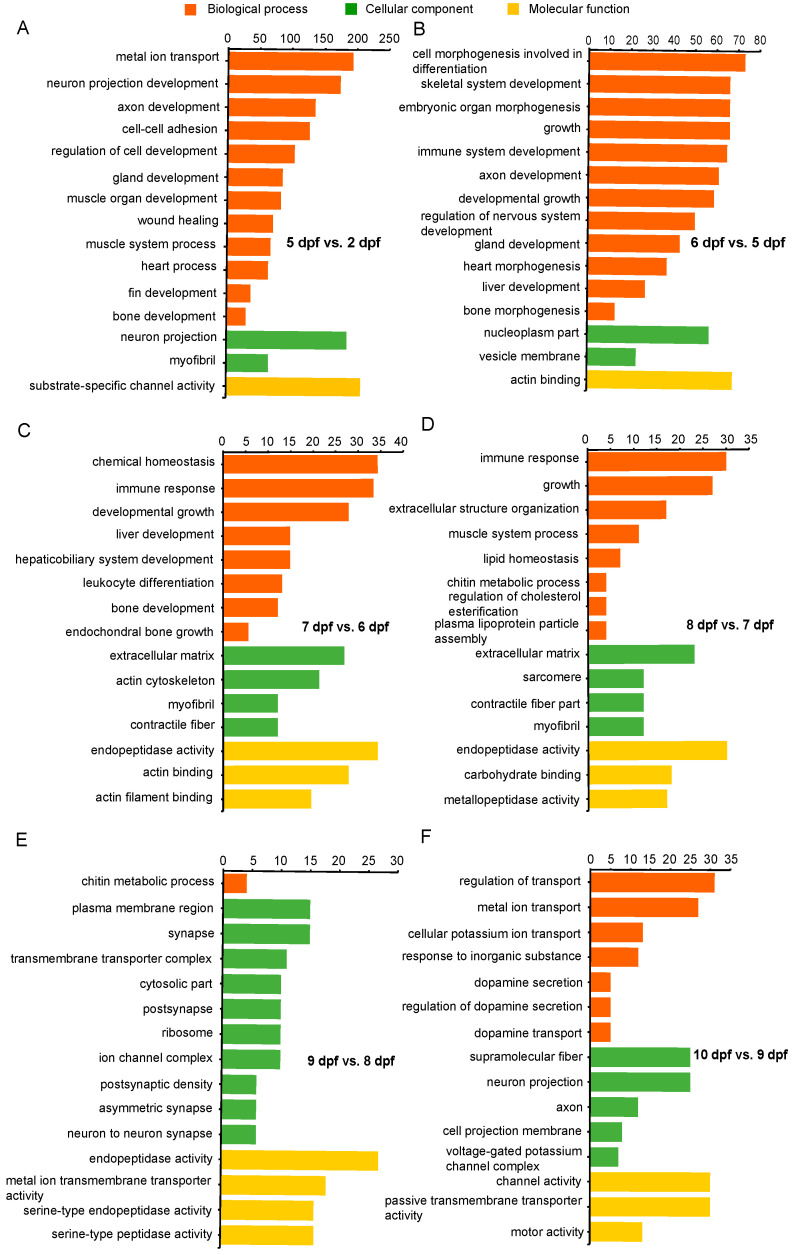
Gene Ontology (GO) functional enrichment analysis of differentially expressed genes (DEGs) at different development stages in channel catfish, *Ictalurus punctatus*. (**A**) 5 vs. 2 dpf, (**B**) 6 vs. 5 dpf, (**C**) 7 vs. 6 dpf, (**D**) 8 vs. 7 dpf, (**E**) 9 vs. 8 dpf and (**F**) 10 vs. 9 dpf. The horizontal axis indicates the number of DEGs between two sampling datasets, and the vertical axis represents the GO terms significantly enriched by the DEGs.

Gene ontology enrichment analysis (reference) of DEGs was conducted for each developmental stage. Significantly enriched categories during channel catfish early development are listed in Supplementary Table S3. The top 15 enriched categories for each stage are listed in Figure 4. Functional annotation of DEGs between 5 and 2 dpf contained categories mainly associated with development, growth, muscle development, regulation of nervous system development, chondrocyte development, bone growth, heart contraction, blood coagulation, fin development and gland development. DEGs between 6 and 5 dpf were mainly enriched in functions pertaining to development, morphogenesis and differentiation. These included genes involving embryonic skeletal system development, connective tissue development, gland development, neuron projection development, immune system development, heart morphogenesis, embryonic organ morphogenesis, cardiocyte differentiation and stem cell differentiation. Between 7 and 6 dpf, enriched categories were most related to development and differentiation that included bone development, liver development, immune response and myeloid leukocyte differentiation. Between 8 and 7 dpf, DEGs were enriched in development, organization and homeostasis activity, such as cardiac muscle fiber development, extracellular matrix organization and immune response. Also, DEGs that were enriched from 9 compared to 8 dpf belong to categories such as synapse activity and ion activity, including postsynaptic specialization, calcium ion transmembrane transporter activity and metal ion transmembrane transporter activity. From Day 9 to Day 10, embryonic gene expression became enriched in selected forms of ion transport, dopamine transport, voltage-gated calcium channels. In addition, neron projectin was enriched. It is notable that during early developmental stages of channel catfish, especially at 5 and 6 days post fertilization (the transition stage from advanced embryo to larva), the most enriched categories were relevant to growth, development, proliferation and morphogenesis.

### 2.5. Construction of Gene Co-Expression Networks

**Figure 5:**
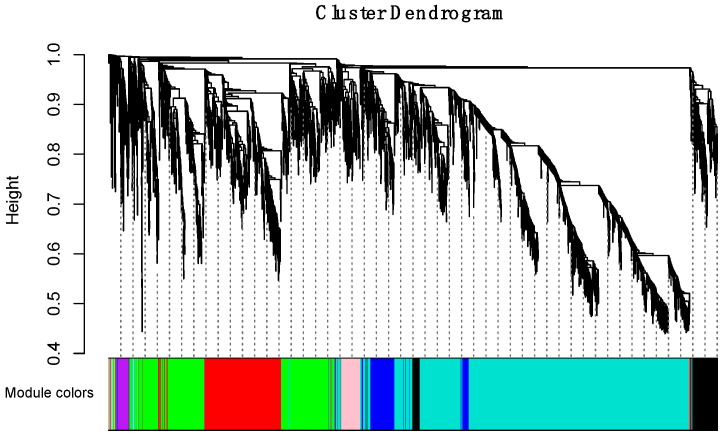
Hierarchical clustering dendrogram of channel catfish, *Ictalurus punctatus*, genes with dissimilarity based on topological overlap during early development. Each single leaf in the tree represents a single gene, the major tree branches constitute 12 distinct modules and are shown in different colors.

**Figure 6:**
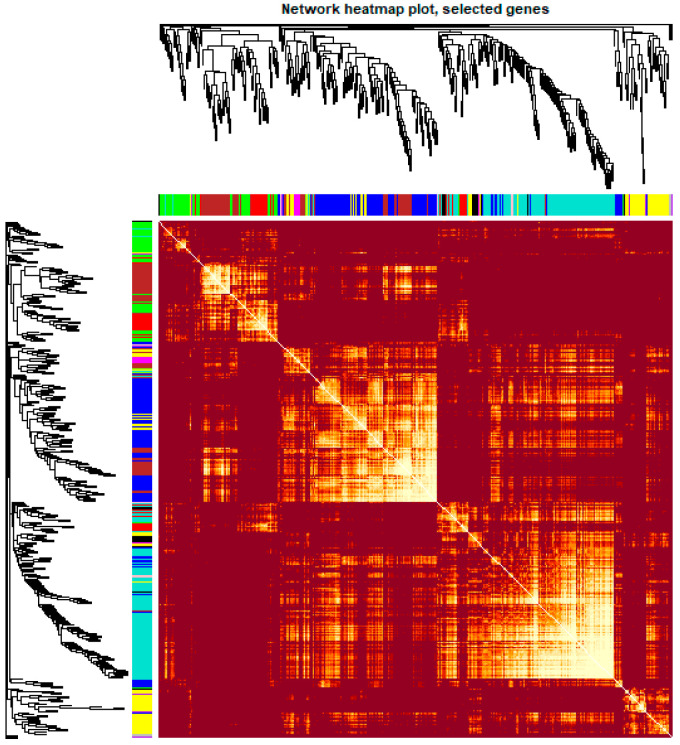
Heatmap plot of the gene network in channel catfish, *Ictalurus punctatus*. The heatmap shows the Topological Overlap Matrix (TOM) among all genes in the analysis. Light color represents high adjacency, and darker color represents low adjacency. The left and top sides indicate the gene dendrogram and module assignment.

To obtain a comprehensive understanding of gene co-expression relationships during development and to characterize the genes that are highly associated with embryogenesis and organogenesis, we applied the WGCNA approach (reference) to the FPKM data resulting from RNA-Seq differential expression analysis. After removing redundant genes, 8504 genes were retained for further WGCNA analysis. The best soft thresholding was determined when the degree of independence was 0.8 (Figure S2). A co-expression network was constructed. This network relys on the assumption that highly cooperating genes were clustered into one module and contributed to the corresponding phenotype. Modules were defined as sets of highly intercorrelated genes, and the genes within same cluster posses a high level of correlation. In total, 12 distinct modules were identified and assigned with different module colors (Figure 5), in which major tree branches define the 12 distinct modules. The 12 modules connected with distinct developmental samples due to sample-specific expression profiles. The interactions of these 12 co-expression modules were analyzed and are shown in Figure 6.

### 2.6. Gene Co-Expression Modules Correspond to Channel Catfish Early Development

**Figure 7:**
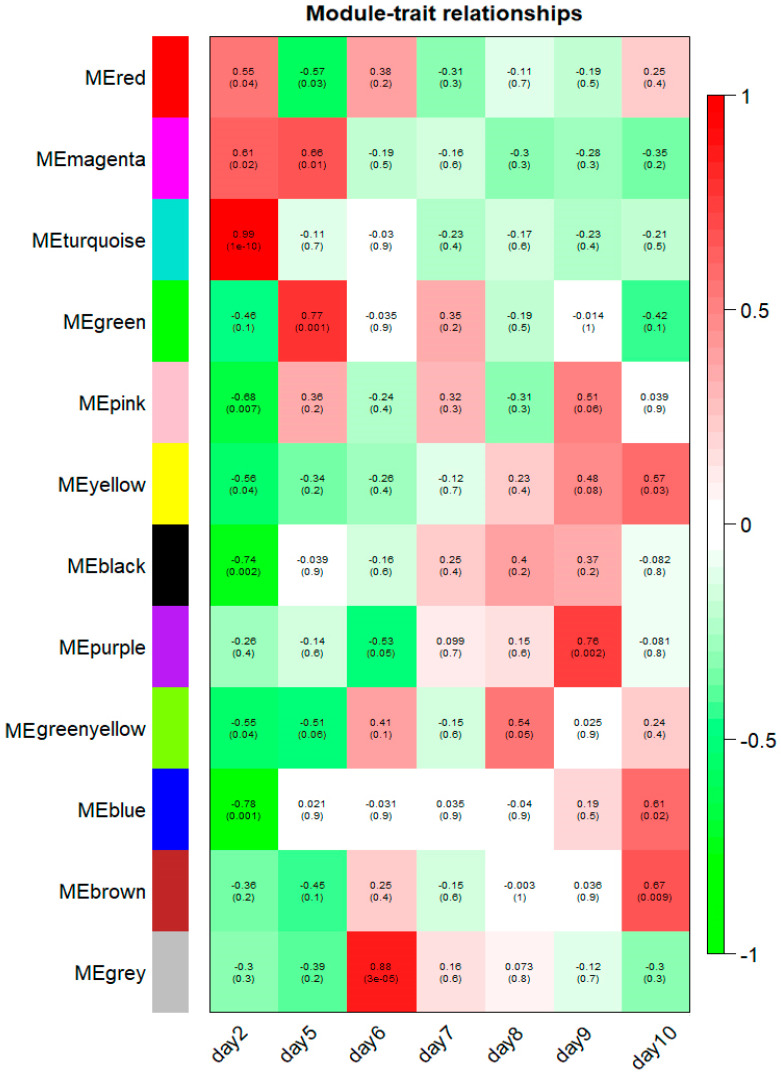
Module-stage relationships (MSRs) in channel catfish, *Ictalurus punctatus*. Each row corresponds to a module, and each column represents a specific development stage. The right color panel represent Pearson’s *r* correlation coefficient. The MSRs are colored based on the correlation coefficient between the module and the developmental stages. The Pearson’s *r* correlation coefficients and associated *p*-values are given in each cell. For better resolution of this figure, the readers are referred to the online version of this figure.

The 12 modules correlated with distinct developmental stages according to their stage-specific profiles, and module-trait associations are shown in Figure 7. Notably, 8 of 12 modules (turquoise, black, blue, pink, green, grey, purple and brown modules) were identified as significantly and highly expressed in one particular sample (*r* > 0.6, *p*-value < 1 × 10^−2^, Figure 7). The turquoise module consists of 2227 genes that were exclusively expressed at the 2 dpf stage (correlation coefficient *r* = 0.99, *p*-value = 1 × 10^−10^). The black, blue and pink modules were negatively correlated with the 2 dpf stage and contain 255; 1916 and 142 genes, respectively (black module: *r* = −0.74, *p*-value = 2 × 10^−3^; blue module: *r* = −0.78, *p*-value = 1 × 10^−3^; pink module: *r* = −0.68, *p*-value = 7 × 10^−3^). The green module contains 910 genes, marking 5 dpf stage-specific expression (*r* = 0.77, *p*-value = 1 × 10^−3^). The grey module, with 22 verified genes, was positively associated with the 6 dpf stage (*r* = 0.88, *p*-value = 3 × 10^−5^). The purple module contains 82 genes that were significantly associated with the 9 dpf stage (*r* = 0.76, *p*-value = 2 × 10^−3^). Also, there were 1174 genes in the brown module corresponding to the 10 dpf stage (*r* = 0.67, *p*-value = 9 × 10^−3^). Correlations between the modules and the developmental traits were quantified and are listed in Table 1, and all genes present in those modules are presented in Supplementary Table S4. 

### 2.7. Functional Enrichment of Genes in the Eight Selected Modules

To investigate the biological functions related to channel catfish in early development, eight modules potentially associated with early developmental stages were selected for Gene Ontology (GO) enrichment analysis (Supplementary Table S4). Significant Gene Ontology (GO) terms in the selected modules were identified using ClusterProfiler R package (version 3.6).

The turquoise module was significantly correlated with the 2 dpf stage. The genes in this module were mainly enriched in four categories: development (muscle cell development, muscle structure development, cell migration involved in heart development, hematopoietic or lymphoid organ development), cell cycle process (cell proliferation, DNA replication, mitotic cell cycle process, meiotic cell cycle process), reproduction process (cellular process involved in reproduction in multicellular organism) and many transport activities (organic acid transmembrane transport, carboxylic acid transmembrane transport, amino acid transport, organic anion transmembrane transporter activity).

The genes in black, blue and pink modules were also strongly correlated with the 2 dpf stage, but they showed a negative correlation relationship with this developmental stage. Genes assigned to the black module were enriched into proteasomal activity (proteasomal ubiquitin-independent protein catabolic process, proteasome complex), endopeptidase complex and endopeptidase activity. Genes in blue module were mainly related to synaptic signaling (chemical synaptic transmission, anterograde trans-synaptic signaling, regulation of trans-synaptic signaling), ion transport (metal ion transport, cation transmembrane transport, calcium ion transmembrane transport), cell and tissue morphogenesis (cell part morphogenesis, cell morphogenesis involved in neuron differentiation, muscle tissue morphogenesis), heart process (regulation of heart rate, heart contraction, regulation of heart contraction) and nervous system development (regulation of nervous system development, nervous system process, positive regulation of nervous system development). Genes in the pink module were mainly associated with cytosolic processes (cytoplasmic vesicle membrane, cytoplasmic vesicle part, cytosolic small ribosomal subunit), and vesicle membrane processes (synaptic vesicle membrane, exocytic vesicle membrane, transport vesicle membrane).

The genes assigned to the green module were significantly correlated with the 5 dpf stage. Genes in this module were enriched in processes of bone development (cartilage development, skeletal system development) and morphogenesis (bone morphogenesis), as well as immune system development (positive regulation of mononuclear cell proliferation, positive regulation of T cell proliferation, positive regulation of lymphocyte proliferation, positive regulation of leukocyte proliferation).

The grey module genes were highly expressed in the 6 dpf stage. This module mainly included Gene Ontology categories of extracellular matrix (extracellular matrix component, collagen-containing extracellular matrix), binding activity (Guanosine-5′-triphosphate (GTP)-binding, purine nucleotide binding, carbohydrate-binding) and symporter activity (potassium:chloride symporter activity, cation:chloride symporter activity, anion:cation symporter activity).

The purple module genes were turned on during the 9 dpf stage. Genes in this module were enriched in the differentiation (regulation of myeloid leukocyte differentiation, regulation of myeloid cell differentiation), homeostasis (cellular calcium ion homeostasis, cellular divalent inorganic cation homeostasis, ion homeostasis) and some transmembrane activity (urea transmembrane transporter activity, water transmembrane transporter activity).

The brown module was significantly related to the 10 dpf stage. Genes in this module were enriched in neuron development (regulation of neuron projection development, neural crest cell development), morphogenesis of different tissues and organs (cell morphogenesis involved in neuron differentiation, cell morphogenesis involved in differentiation, sensory organ morphogenesis, dendrite morphogenesis, inner ear morphogenesis).

The enriched terms for these stage-specific co-expressed modules were in agreement with our previous GO enrichment analysis and morphology observations, which belong to tissues and organ differentiation, regulation of ion transportation, cell proliferation and transmembrane activities.

### 2.8. Protein-Protein Interaction Network Construction and Analysis of Selected Modules

**Figure 8:**
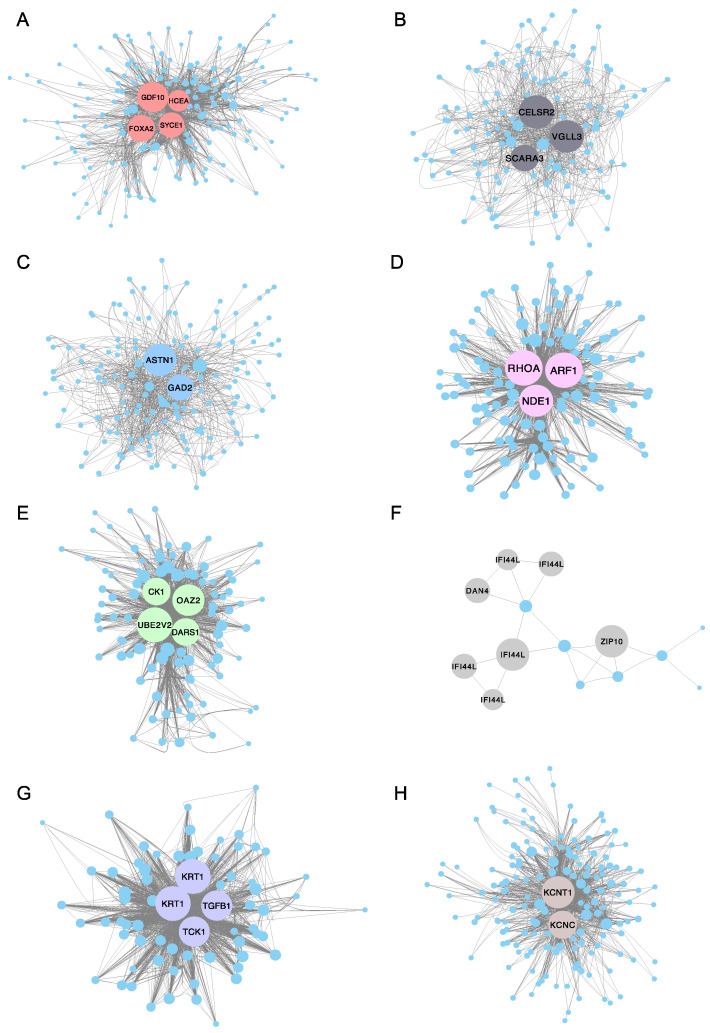
Protein–protein interaction (PPI) network for eight interested modules in channel catfish, *Ictalurus punctatus*, predicted by Cytoscape. The node degree of genes was represented using circumference of nodes. The genes with red, green, grey or purple color represent the hub genes in the (**A**) turquoise module, (**B**) black module, (**C**) blue module, (**D**) pink module, (**E**) green module, (**F**) grey module, (**G**) purple module and (**H**) brown module, separately.

To further identify the function of the co-expressed genes within each module and investigate the hub genes, Cytoscape (version 3.7.2) software was used to construct a co-expression network of the top 200 ranked genes for eight selected modules (Supplementary Table S5), including the turquoise, green, grey and purple modules. Notably, the pink, grey and purple modules only have 142, 22 and 82 genes separately, so all genes in these three modules were selected for co-expression network construction. The highest degree genes (hub genes) were illustrated with a bigger size and specific color (Figure 8). For example, *GDF10* (growth/differentiation factor 10-like), *FOXA2* (hepatocyte nuclear factor 3-beta-like), *HCEA* (high choriolytic enzyme 1-like) and *SYCE3* (synaptonemal complex central element protein 3) were identified as hub genes in the turquoise module for the 2 dpf stage. *CK1* (casein kinase I), *DARS1* (aspartyl-tRNA synthetase 1), *UBE2V2* (ubiquitin conjugating enzyme E2 variant 2) and *OAZ2* (ornithine decarboxylase antizyme 2-like) were identified as hub genes in the green module (correlated with the 5 dpf stage). In the grey module, *IFI44L* (interferon-induced protein 44-like) and *ZIP10* (zinc transporter 10-like) were recognized as hub genes (correlated with the 6 dpf stage). *TGFB1* (transforming growth factor beta-1-like) and *TCK1* (creatine kinase, testis isozyme-like) were verified as hub genes in the purple module (correlated with the 9 dpf stage). Similarly, *KCNT1* (potassium channel subfamily T member 1-like) and *KCNC* (potassium voltage-gated channel subfamily C member 1) were identified as hub genes in the brown module (correlated with the 10 dpf stage). A complete list of hub genes and their descriptions are shown in Table 2.

## 3. Discussion

Gene expression changes are complex during the transition from fertilized eggs to larvae, and the transcriptome profiles underlying these events have not been fully studied in catfish. The main objectives of this study were to utilize a transcriptome sequencing method to analyze the expression of seven early developmental stages in channel catfish and to construct a gene co-expression network involved in embryogenesis. In this study, we investigated a set of DEGs in each developmental stage by comparing each of the two successive stages and found that the highest numbers of DEGs occurred at 5–7 days post fertilization. The vast differences in transcript expression illustrate that these stages are of rapid, critical, expansive development in channel catfish in accordance with morphological change observation. Gene Ontology enrichment analysis revealed that during early embryogenesis, the most enriched Gene Ontology categories were related to development, growth, differentiation and morphogenesis, especially during 5–7 dpf. These stages are critical for the development of muscle, nerves, bone and other tissues, and cell differentiation during transformation from fertilized eggs to larvae.

WGCNA [17] is a powerful method to identify co-expressed groups of genes from large RNA expression datasets [21], and is widely used to explore the correlation among transcriptomic datasets, identify hub genes and discover new pathways in both model and non-model species [22,23,24]. WGCNA has proven its superiority over partial correlation methods and provides a powerful tool for identifying higher-order correlation in complex traits of interest, by presenting a simplified network on the integrated function of gene modules [25,26].

Our study is the first systematic characterization of catfish early development at the transcriptome level. Previous research mainly focused on the physical development and anatomy. The first study related to the embryonic development of channel catfish was conducted from 2 dpf through 11 dpf, with a focus on the organogenesis of pronephros [7,8]. Later on, a more detailed description was performed at 1 h intervals after fertilization for a 34 h period, which corresponded to the emergence of the pectoral fin-buds at water temperatures from 24.7 to 26.8 °C [6]. Specific developmental stages of channel catfish embryos were for the first time defined based on the development of the vascular system at 26 °C. All the stages were characterized before 5 dpf [27]. Makeeva et al. reported that stage IV (1 dpf) was mainly associated with gastrulation, stage V (~1.5 dpf) related to organogenesis and stage VI (~2 dpf) resulted in formation of gill microstructure, eye lens, auditory vesicles and segmentation of tail. At the end of the second day of development, they observed the heartbeat, and also the rotation of embryo within the egg case, with its tail whipping back and forth. Stage VII (~3 dpf) was associated with development of the vascular system, development in the brain and an intestinal cavity can also be observed at this stage [27]. In general, our observations agreed well with those of earlier studies. The functional annotation of DEGs between 5 and 2 dpf revealed that the enriched terms were most associated with muscle tissue development, bone growth, morphogenesis of different organs, chondrocyte development, heart contraction, blood coagulation and regulation of blood circulation. However, our morphological observations found that the embryonic tails were not observed until 5 dpf, which is slightly later than previous observations for channel catfish embryonic development [27]. Our fertilized eggs were incubated at 25 to 26 °C (mainly 25 °C), which is not that much different than earlier studies. Perhaps, the slower development for our channel catfish was due to the strain, water quality or oxygen levels, which could lead to variation in the speed of development [27].

In the current study, and for the first time, a gene co-expression network was used to investigate the transcripts of the embryos and fry in different early developmental stages of channel catfish. Using contrasted biological samples at different developmental stages of channel catfish embryos, four distinct modules were identified. The genes within the same intra-module were used to perform Gene Ontology enrichment analysis, and the genes most associated with 2, 5, 6 and 9 dpf stages were identified as enriched in development, proliferation, morphogenesis and differentiation categories, such as muscle structure development, hematopoietic or lymphoid organ development, bone morphogenesis and regulation of myeloid cell differentiation categories. Then, Cytoscape was employed to build a PPI network for the four selected modules, and the high degree of genes (hub genes) was verified to have an essential role in the co-expression network.

### 3.1. Turquoise Module (Hub Genes and Node Genes)

The module most associated with the 2 days post-fertilization stage was the turquoise module, which was a critical first stage for the transition of fertilized egg to an adult-like fry. The hub genes were *GDF10*, *FOXA2*, *HCEA* and *SYCE3*. *GDF10* is a growth differential factor belonging to the TGC-beta (transforming growth factor beta) superfamily, and functions predominantly in bone development [28]. Its pathways are p70S6K signaling and activation of cAMP-dependent protein kinase A [29,30]. *GDF10* is necessary for head formation, skeletal morphogenesis and adipogenesis [31,32,33].

*FOXA2* is a transcription factor involved in embryonic development [34], and regulation of gene expression in differentiated tissues and development of multiple endoderm-derived organ systems, such as liver, gland, pancreas and lungs [35,36,37,38]. This gene is related to pathways that include heart developmental, Hedgehog signaling events mediated by Gli proteins and cardiac progenitor differentiation [29,30].

*HCEA* participates in the breakdown process of the egg envelope, which is derived from the egg extracellular matrix [39,40]. *HCEA* has a typical neutral zinc metallopeptidase domain that is involved in the binding of zinc and proteolysis [41].

Another candidate hub gene for the 2 dpf module is *SYCE3*, which has associated pathways that include the cell cycle, mitosis and meiosis [29,30]. *SYCE3* is a significant component of the transverse central element of synaptonemal complexes (SCS) formed between homologous chromosomes during meiotic prophase. This gene is also required for chromosome loading of the central element-specific SCS proteins, and for initiating synapsis between homologous chromosomes as well as being required for fertility [42,43]. These hub genes findings, together with GO enrichment results in the turquoise module, imply that the 2 dpf stage is a critical time window related to tissue differentiation, morphogenesis and different organ development, such as bone morphogenesis, head formation, skeletal morphogenesis, live development, gland and lungs development, which requires a series of concerted meiosis, mitosis and synapsis activity, and *SYCE3* may play an essential role regulating early development through these functions.

Gene names and gene IDs of the remaining 2223 genes in the turquoise module are listed in Supplementary Table S3. The functional characterization of these genes in the zebrafish model can be retrieved from the ZFIN (Zebrafish Information Network) [44].

### 3.2. Black Module

The black module was most associated with 2 dpf, hub genes in this module included *VGLL3*, *CELSR2* and *SCARA3*. The VGLL proteins are transcriptional co-factors influencing myogenesis in skeletal muscle. Figeac et al. reported that VGLL3 could contribute to muscle fiber composition in mice, and knockout of *VGLL3* gene in mice suppressed myoblast proliferation. Conversely, the overexpression of *VGLL3* highly increased myogenic differentiation. This research stated that *VGLL3* was a transcriptional co-factor working with the Hippo signal transduction to control myogenesis [45]. Also, *VGLL3* was verified to be linked with age at maturity in Atlantic salmon (*Salmo salar*). *VGLL3* and the interrelated Hippo pathway has been reported to be linked to the decreased proliferation process in different tissues, and might play a negative role on Sertoli cell proliferation in testis, thus, compressing the growth of pubertal testis [46].

*CELSR 1-3* expression started broadly in the nervous system in early developmental stages. These genes were found to be expressed in other organs, such as the cochlea, the kidney and the whisker. *CELSR2* protein was distributed along intercellular boundaries in the whisker and related to neuronal cells [47]. Also, the *CLESR2* gene was reported to play a role in the regulation of facial motor neurons’ migration in the neuroepithelium during the development of the zebrafish hindbrain [48].

*SCARA3* belongs to the class A scavenger receptors (SR-As) family, which was identified to be functioning with the innate immunity in mammals [49]. This gene was also identified to potentially contribute to immunity in rainbow trout (*Oncorhynchus mykiss*) [49]. Further research is needed to see if this gene also functioned as an immunity regulator in all teleost fish.

### 3.3. Blue Module

For the blue module, which was most correlated with 2 dpf, hub genes contained *ASTN1* and *GAD2*. The *ASTN1* works as a neuron-glial ligand for central nervous system glial-guided migration [50]. *ASTN1* is necessary for normal migration of young post-mitotic neuroblasts along glial fibers. It also plays an important role for the migration of granule cells during brain development [50,51]. In zebrafish, *GAD2* plays a role in the dorsal hindbrain development [52]. *GAD2* is also involved in the neurotransmitter release cycle and beta-alanine metabolism pathway [29].

### 3.4. Pink Module

For the pink module, which was most correlated with 2 dpf, hub genes were: *ARF1*, *NDE1* and *RHOA*. *ARF1* was considered to be coupled with *CDC42* to regulate the endocytosis in the plasma membrane [53]. The association of ARF1 and endosomal membranes was regulated by the endosomal pH, which controls the pH-dependent association of endosomal Coat proteins (COPs). ARF1 could influence COP function through the endocytic pathway, which may suggest that ARF1 might act as the cytosolic component for a transmembrane pH-sensing mechanism.

*NDE1* was reported to play an essential role for centrosome duplication and mitotic spindle assembly. The function and orientation of the mitotic spindle was critical for normal cerebral cortex development in mammals [54,55]. *RHOA* encodes a member of the Rho family of small GTPases, which could promote the reorganization of the actin cytoskeleton as well as regulate the shape and motility of organisms [56]. All these findings indicate that appropriate gene expression at 2 dpf was vital for the survival of individual as well as the development and function of different organs. More detailed study of early embryonic development, tracing the formation of a specific tissue/organ, or even the expression and migration of a specific gene at 2 dpf, should be examined in the future.

### 3.5. Green Module

For the green module, which was most correlated with 5 dpf, its hub genes included *CK1*, *DARS1*, *UBE2V2* and *OAZ2*. The CK1 family of protein kinases are serine/threonine-selective enzymes, which function as key regulatory molecules involved in the cell cycle, DNA repair, transcription, translation, the structure of the cytoskeleton, cell–cell adhesion and receptor-coupled signal transduction [57,58]. CK1 is also involved in the Wnt signaling pathway [29]. The Wnt signaling pathway plays an important role in lung organogenesis, the initial formation of the neural plate and many subsequent patterning decisions in the embryonic nervous system [59,60]. The Wnt signaling pathway also works with other signaling systems as primary molecular mechanisms that control embryonic development, and regulate processes such as cell proliferation, survival, or differentiation [61].

*DARS1* is critical to the processes of tRNA aminoacylation, selenoamino acid metabolism as well as gene expression [29,30]. The *DARS1* gene encodes a member of a multienzyme complex, which catalyzes the attachment of an amino acid (AA) to its connate tRNA in a two-step reaction. The amino acid is first activated by ATP to form AA-AMP and then transferred to the acceptor end of the tRNA [62]. Although *DARS1* is considered to be expressed in all organs, it has a distinct expression pattern in the brain. Dars-knockout mice were not viable and displayed early developmental arrest with associated embryonic lethality [63,64]. Mutations of *DARS1* and its homolog *DARS2* have been reported in patients showing hypomyelination in the brainstem, spinal cord and leg spasticity (HBSL), and leukoencephalopathy brain stem and spinal cord involvement and elevated lactate (LBSL), which demonstrates that mutation in tRNA causes a similar disease and shares a common mechanism of neurological pathology [65].

*UBE2V2* is thought to be involved in the differentiation of monocytes and enterocytes [66], and it may also play a role in progression through the cell cycle, as well as differentiation [43,67]. Among its related pathways are DNA double-strand break repair, and class I MHC-mediated antigen processing and presentation [29,30].

*OAZ2* plays a role in cell growth and proliferation by regulating intracellular polyamines [43,68]. Its related pathways are cyclin-dependent kinase (CDK)-mediated phosphorylation, removal of cdc6 and metabolism [29,30]. Five dpf is considered as a transition period from fertilized egg to adult-like fry, and the larvae possess movement at this stage. The hub genes identified at this stage were correlated with regulation of the cell cycle and transcription activities, as well as formation of the nervous system.

### 3.6. Grey Module

Another module of interest was the grey module, which was most strongly associated with 6 dpf. Hub genes in this module included *IFI44L* and *ZIP10*. *IFI44L* is reported to be associated with virus infection and immune activity, as well as the formation of microtubular structures [69,70,71]. This gene has not been verified to be closely related to early embryonic development; however, the PPI network predicts that *IFI44L* plays an essential role in this module, illustrating that *IFI44L* may be related to early immune response and the survival of embryos, and thus, contributes to the early development mechanisms. The other hub gene in this module is *ZIP10*, which controls the membrane transport of zinc, calcium, manganese and regulates their intracellular and cytoplasmic concentrations [72].

Functions of most other node genes, i.e., *SLC12A8*, *MTFR1L*, *Tatdn2*, *Agxt*, *MX2*, *nd4l* and *nd6*, have not been experimentally documented in embryogenesis and somitogenesis of fish [44]. *SLC12A8* (solute carrier family 12 member 8) was an important paralog gene of *SLA12A2*, Gene Ontology (GO) annotations assign this gene possible function that includes ATPase activity, coupled to transmembrane movement of substances and symporter activity. *MTFR1L* (Mitochondrial Fission Regulator 1 Like) was a paralog of *MTFR2*, which may play a role in mitochondrial aerobic respiration in the testis. It also promotes mitochondrial fission. *MX2* (MX dynamin like GTPase2) was involved in the innate immune system [44].

The related pathways of *Tatdn2* (TatD DNase Domain Containing 2) are unfolded protein response (UPR) and metabolism of proteins. The GO annotations related to this gene include deoxyribonuclease activity and endodeoxyribonuclease activity. Agxt (alanine-glyoxylate and serine-pyruvate aminotransferase) are predicted to have alanine-glyoxylate transaminase activity and serine-pyruvate transaminase activity. Both nd4l (ND4L-NADH dehydrogenase, subunit 4L) and nd6 are involved in respiratory electron transport, ATP synthesis by chemiosmotic coupling and heat production by uncoupling proteins [44]. At 6 dpf, channel catfish larvae possess relatively full swimming ability. One of the most important results at 6 dpf came from GO enrichment and PPI network analysis, which indicated that this stage was mainly associated with early immune response, shedding light for further study of immune mechanisms in channel catfish or even other teleost fish.

### 3.7. Purple Module

The purple module was correlated with 9 dpf. This module contained *TGFB1* and *TCK1*. TGFB1 can regulate cell proliferation, differentiation of various cell types and function in other processes such as normal development, immune function and responses to neurodegeneration [43,73,74]. Among its related functions are transcription androgen receptor nuclear signaling and the p70S6K signaling pathway [29,30].

TCK1 reversibly catalyzes the transfer of phosphate between ATP and various phosphagens, and also plays a crucial role in tissues with high energy requirements, such as in skeletal muscle, heart, brain and spermatozoa [43,75,76].

The other node genes (Supplementary Table S3), *AQP7*, *AQP8*, *cldn11*, *MMP9* and *clec14a* may be involved in the somitogenesis and normal function of organs during early development. Zebrafish *AQP7* (Aquaporin 7) was maternally inherited and detected at the 2–4 cell and morula stages [77], while other paralogs such as *AQP8aa* were related to somitogenesis and vascular development [78]. *Cldn11a* (Claudin 11), expression was detected in vascular endothelium, adjacent to the optic stalk of embryo in normal retinal embryo [79,80]. *cldn11* expression requires *zic2* function in the differentiating mammalian cerebellar ganglion cells [81]. *MMP-2* (matrix metallopeptidase 2), *MMP-9* and *MMP-13* were necessary for proper zebrafish craniofacial morphogenesis as morpholino knockdown of these genes shows huge alterations in both the mandibular and hyoid arches concurrently [82]. By interacting with *Etv2* and *Vegf* signaling, *Clec14a* (C-lectin family 14 member A) in zebrafish was indispensable for function of vascular endothelia cells during vasculogenesis and angiogenesis, as knockdown of *Etv2*/*Vegf* results in an inhibition of intersegmental vessel sprouting [83]. The 9 dpf channel catfish larvae was more like an adult fish and possesses fully swimming ability. Our findings reveal that this stage mainly related to the process of immune function, neurodegeneration and vascular development.

### 3.8. Brown Module

The last module is the brown module, which was correlated with 10 dpf. The hub genes in this module contain *KCNT1* and *KCNC*. The *KCNT1* gene belongs to the potassium channel family, which is considered to regulate ion flux. It could also interact with cytoplasmic proteins related to developmental signaling pathways [84]. The *KCNC* gene belongs to a potassium voltage-gated channel family, which was critical for the rapid repolarization of fast firing brain neurons [29]. In response to the voltage across the membrane, the channel opens, forming a potassium-selective channel, and potassium ions, in accordance with their electrochemical gradient, could cross the channel [29,85]. The period of 10 dpf was the last timepoint selected in this channel catfish early development study as at this time, the fry can swim freely and possess relative complete function.

## 4. Materials and Methods

### 4.1. Ethics Statement

All of the experimental protocols involved in animal care and sample collection were approved by the Auburn University Institutional Animal Care and Use Committee (AU-IACUC, PRN#: 2016-2901 on 13 June 2016). All samples were collected after euthanization with buffered MS-222 (200 mg/L). All animal handling procedures were performed following the Guide for the Care and Use of Laboratory Animals and the Animal Welfare Act in the United States.

### 4.2. Sampling of Channel Catfish

The Kansas random strain of channel catfish was raised in earthen ponds at the Genetics Research Unit, E.W. Shell Research Center, Auburn University. The Kansas strain was derived from the Ninnescah River in Pratt, Kansas, in 1911 [86,87], and is the oldest domestic channel catfish strain in the US.

After harvesting, females and males were mated and spawned at the genetics facility greenhouse. Channel catfish embryos and larvae were obtained by artificially spawning brood stock. Fertilized eggs were incubated in a hatching trough at 25 to 26 °C, with water hardness above 40 ppm and dissolved oxygen level of 5 mg/L. Treatments ceased 24 h before the expected hatch date. From 7 dpf, swim-up fry were fed to satiation six times per day using a powdered 50% protein starter diet from Purina^®^ AquaMax^®^ (Purinamill, Arden Hills, MN, USA) [87]. To contain a large timeframe of channel catfish samples, including stages of fertilized egg to a mature-like fry, channel catfish samples were obtained at 2, 5, 6, 7, 8, 9 and 10 dpf based on their developmental stages. A total of 20–50 embryos or larvae were collected at each sampling, and 200 ppm buffered MS-222 was utilized to euthanize the larvae [87]. The samples were placed into 1.5 mL centrifuge tubes, flash-frozen in liquid nitrogen and stored in −80 °C for RNA extraction.

### 4.3. Microscopic Anatomy

At each sampling timepoint, another 20–50 embryos/larvae were fixed with 10% phosphate-buffered formalin in 1.5 mL centrifuge tubes and sealed for microscopic analysis. For microscopic anatomical observations, samples were transferred to a 75% ethanol solution. Observations were conducted with a MEIJI TECHNO anatomy microscope (MEIJI Techno America, San Jose, CA, USA) and images were photographed with a Canon DS126311 camera.

### 4.4. RNA Isolation, Library Construction and Sequencing

In order to examine changes in expression throughout early stages of development, at each time point, 2, 5, 6, 7, 8, 9 and 10 dpf, eight embryos/larvae were randomly selected and divided into two replication pools (four embryos or larvae each). For each replicate, samples of four embryos/larvae were homogenized in liquid nitrogen with a mortar and pestle. RNA extraction was conducted using a Qiagen RNeasy Plus Kit following the manufacturer’s protocols. The concentration and purity of RNA samples were measured using a NanoDrop spectrophotometer (Thermo Scientific, Madison, WI, USA). For each time point, equal amounts of RNA from the two pooled replicates were used for library construction and Illumina RNA sequencing [88].

Library construction and sequencing reactions were conducted at GENEWIZ, LLC. (South Plainfield, NJ, USA). The RNA integrity was checked with 4200 TapeStation (Agilent Technologies, Palo Alto, CA, USA). Ribosomal RNA depletion was conducted using Ribozero rRNA Removal Kit (Illumina, San Diego, CA, USA). RNA sequencing library preparation was performed using a NEBNext Ultra RNA Library Prep Kit for Illumina (NEB, Ipswich, MA, USA). Enriched RNAs were fragmented for 15 minutes at 94 °C. The first strand and second strand cDNA were subsequently synthesized. The cDNA fragments were end-repaired and adenylated at 3’ ends, and a universal adapter was ligated to cDNA fragments, followed by index addition and library enrichment with limited cycle PCR. Sequencing libraries were validated using the Agilent Tapestation 4200 (Agilent Technologies, Palo Alto, CA, USA) and quantified by using a Qubit 2.0 Fluorometer (Invitrogen, Carlsbad, CA, USA). The samples were sequenced using a 2 × 150 paired-end (PE) configuration. Image analysis and base calling were conducted by the HiSeq Control Software (HCS). The raw RNA-seq data is available at NCBI GEO (Gene Expression Omnibus) databases under the accession number GSE152002.

### 4.5. Reads Mapping and Differential Expression

The channel catfish reference genome was used for mapping [14]. Channel catfish transcriptome data (.bcl files) generated from Illumina HiSeq were converted into fastq files and de-multiplexed using Illumina’s bcl2fastq software (version 2.17). The quality of raw reads was controlled by FASTQC [89]. Raw reads were filtered by removing low-quality reads, adapters and reads with length shorter than 36 bases using Trimmomatic v0.36 [90]. The resulting clean reads were quality-controlled again and aligned to the channel catfish genome by STAR software (version 2.7.0) [91], allowing less than a 4-bases mismatch. HTSeq-count [92] was conducted to calculate the number of aligned reads of each gene overlapping its exons. To perform the differential expression analysis with the embryonic development of channel catfish, an R package DESeq2 [93] was employed to calculate the log2-fold change (log2FC), a criterion with |log2FC| ≥ 1, and adjusted *p*-value < 0.05 was used as the threshold for evaluating the DEGs. The log2FC > 1 DEGs were considered to be upregulated, while log2FC < −1 DEGs were considered to be downregulated.

### 4.6. Enrichment Analysis

For every differential expression comparison, the Gene Ontology (GO) terms of each gene were assigned by using zebrafish annotations for the unigene set. Enrichment analysis was also performed using the ClusterProfiler R package (version 3.6) [94] to profile their major biological processes, molecular functions and cellular components. The threshold of significance criteria was set at a 0.05 *q*-value cutoff and the enriched GO terms were ranked by *p*-value.

### 4.7. Gene Co-Expression Network Construction

To verify the interesting gene modules and network properties of the gene expression profile in the early development of channel catfish, an R package named weighted gene co-expressed network analysis (WGCNA) [17] was employed following the standard protocol. Then, the intramodular connectivity and gene significance were applied to verify key co-expressed genes in the network and correlate the identified modules to external information development stages. A total of 8504 DEGs from 7 timepoints were used to calculate the correlation between samples and for the hierarchical clustering analysis.

### 4.8. Identification of Development-Related Modules and Visualization

After the co-expression network was constructed, the developmental stage-responsive modules and genes were selected based on the correlation coefficient between modules and developmental stages. The genes within the same modules are highly connective, and the genes inside the same module potentially have similar functions. To verify the biological function of the specific modules as well as the correlation between the modules and different development stages, genes inside the same modules were selected to perform GO enrichment analysis using the R package ClusterProfiler (version 3.6) [94]. Hub genes are defined as genes inside co-expression modules with high correlation. To further verify the hub genes and their possible roles in early development stages, the top 200 ranked genes within each module were extracted according to the intra-modular connectivity with module eigengenes, which were used to construct a protein–protein interaction (PPI) network. The R package Cytoscape (version 3.7.2) [95] was employed to identify genes of the highest node degree, which may have a critical function in the PPI.

## 5. Conclusions

In conclusion, a comprehensive transcriptome analysis was used to explore the dynamic changes during channel catfish early development. This study provides genomic data that has great promise for improved understanding of channel catfish embryogenesis. A co-expression network was constructed using the WGCNA method. As a result of that effort, 12 modules were verified, eight of them were identified to be closely associated with channel catfish early development. Further analysis of these eight selected modules revealed that they were enriched in biological processes related to development, morphogenesis, growth and differentiation. Five- and six-days post-fertilization embryos contained the most strongly differentially expressed genes (DEGs). Gene Ontology (GO) analysis revealed that enriched categories at 5 and 6 dpf were most related to embryonic development, morphogenesis, differentiation and formation of different organs. In addition, these stages display the most striking changes in morphology. Thus, day 5 and 6 are likely to be critical turning points in the progression from fertilized egg to larva in channel catfish. Hub genes identified within each module are likely to direct critical roles during the development and growth processes in channel catfish. Taken together, our results are a first stage in shedding light on the complex biological processes that take place during early development. Our work provides a useful genetic resource for future studies on the metabolism, growth and genetic enhancement programs of channel catfish. Further research should address gene quantification and genetic behavior. Gene editing technology will be used to confirm the function of these genes in WGCNA network.

## Figures and Tables

**Table 1:** Significant co-expression modules in channel catfish development.

Module Color	Number of Genes	Correlation (*r*)	*p*-Value
Turquoise	2228	0.99	1 × 10^−10^
Black	255	−0.74	2 × 10^−3^
Blue	1916	−0.78	1 × 10^−3^
Pink	142	−0.68	7 × 10^−3^
Green	910	0.77	1 × 10^−3^
Grey	22	0.88	3 × 10^−5^
Purple	82	0.76	2 × 10^−3^
Brown	1173	0.67	9 × 10^−2^

**Table 2:** List of hub genes in channel catfish development co-expression modules.

Module	Gene ID	Gene Name	Description
Turquoise	108262421	*GDF10*	Growth/differentiation factor 10-like
Turquoise	108257557	*FOXA2*	Hepatocyte nuclear factor 3-beta-like
Turquoise	108259527	*HCEA*	High choriolytic enzyme 1-like
Turquoise	108268486	*SYCE3*	Synaptonemal complex central element protein 3
Black	108266139	*VGLL3*	Vestigial like family member 3
Black	108281038	*CELSR2*	Cadherin EGF (epidermal growth factor) LAG (laminin G) seven-pass G-type
Black	108258146	*SCARA3*	Receptor 2-likescavenger receptor class A member 3
Blue	108267173	*ASTN1*	Astrotactin 1
Blue	108272908	*GAD2*	Glutamate decarboxylase 2
Pink	108255676	*ARF1*	ADP-ribosylation factor 1-like
Pink	108277500	*NDE1*	Nude neurodevelopment protein 1
Pink	108254710	*RHOA*	Transforming protein rhoa-like
Green	108274016	*CK1*	Casein kinase I
Green	108266474	*DARS1*	Aspartyl-trna synthetase 1
Green	108264029	*UBE2V2*	Ubiquitin conjugating enzyme E2 variant 2
Green	108279393	*OAZ2*	Ornithine decarboxylase antizyme 2-like
Grey	108266103	*IFI44L*	Interferon-induced protein 44-like
Grey	108264165	*ZIP10*	Zinc transporter 10-like
Purple	108279384	*TGFB1*	Transforming growth factor beta-1-like
Purple	108262114	*TCK1*	Creatine kinase, testis isozyme-like
Brown	108256037	*KCNT1*	Potassium channel subfamily T member 1-like
Brown	108275314	*KCNC*	Potassium voltage-gated channel subfamily C member 1

## Supplementary Materials

The following supplementary materials can be found at https://www.mdpi.com/1422-0067/21/15/5535/s1: Table S1: Summary statistics of the transcriptome sequencing in channel catfish (*Ictalurus punctatus*) early development stages (CCN1-N2; channel catfish days post-fertilization (dpf) replicate number). Table S2: Normalized gene read counts in Channel catfish across 7 developmental stages. Table S3: Differentially expressed genes (DEGs) in Channel catfish across 7 developmental stages. Table S4: A list of genes in eight selected modules and Gene Ontology enrichment analysis. Table S5: A list of genes for Cytoscape analysis in each module. Figure S1: Overview of RNA-Seq mapping in channel catfish, *Ictalurus punctatus*, early development. N1dpf-N2: channel catfish days post-fertilization (dpf) replicate number. Figure S2: Analysis of network topology for different soft thresholding powers in channel catfish, *Ictalurus punctatus*. In the left diagram, y-axis is the scale-free fit index as a function of the soft-thresholding power (x-axis). The right diagram shows the mean connectivity (degree, y-axis) as a function of the soft-thresholding power (x-axis).

## Abbreviations

RNA-Seq	RNA-sequencing
WGCNA	Weighted gene co-expressed network analysis
FPKM	Fragments per kilobase of exon model per million reads mapped
WGS	Whole-genome sequencing
DEGs	Differentially expressed genes
PE	Paired-end
AA	Amino acid
log2FC	Log2-fold change
DPF	Days post fertilization
GO	Gene ontology
PPI	Protein–protein interaction network
HCS	HiSeq Control Software
SCS	Synaptonemal complexes
ZFIN	Zebrafish Information Network
MSRs	Module-stage relationships

## References

[1] Eschmeyer W.N., Fricke R., Fong J.D., Polack D.A. (2010). Marine fish diversity: History of knowledge and discovery (Pisces). Zootaxa.

[2] Tan Y., Chang S.K. (2018). Isolation and characterization of collagen extracted from channel catfish (*Ictalurus punctatus*) skin. Food Chem..

[3] Jiang H., Wang M., Fu L., Zhong L., Liu G., Zheng Y., Cheng X., Bian W. (2020). Liver transcriptome analysis and cortisol immune-response modulation in lipopolysaccharide-stimulated in channel catfish (*Ictalurus punctatus*). Fish Shellfish Immunol..

[4] Zeng Q., Liu S., Yao J., Zhang Y., Yuan Z., Jiang C., Chen A., Fu Q., Su B., Dunham R. (2016). Transcriptome display during testicular differentiation of channel catfish (*Ictalurus punctatus*) as revealed by RNA-Seq analysis. Biol. Reprod..

[5] Yang Y., Fu Q., Liu Y., Wang X., Dunham R., Liu S., Bao L., Zeng Q., Zhou T., Li N. (2018). Comparative transcriptome analysis reveals conserved branching morphogenesis related genes involved in chamber formation of catfish swimbladder. Physiol. Genom..

[6] Saksena V.P., Riggs C.D., Yamamoto K. (1961). Early development of the channel catfish. Progress. Fish-Cult..

[7] Wellner K. The Development of the Pronephros during the Embryonic and Early Larval Life of the Catfish (*Ictalurus punctatus*) (1932), by Rachel L. Carson. https://hpsrepository.asu.edu/handle/10776/7567.

[8] Carson R.L. (1932). The Development of the Pronephros During the Embryonic and Early Larval Life of the Catfish (*Ictalurus punctatus*). Master’s Thesis.

[9] Tucker C.S., Hargreaves J.A. (2004). Biology and Culture of Channel Catfish.

[10] Vesterlund L., Jiao H., Unneberg P., Hovatta O., Kere J. (2011). The zebrafish transcriptome during early development. BMC Dev. Biol..

[11] Sharov A.A., Piao Y., Matoba R., Dudekula D.B., Qian Y., VanBuren V., Falco G., Martin P.R., Stagg C.A., Bassy U.C. (2003). Transcriptome analysis of mouse stem cells and early embryos. PLoS Biol..

[12] Hartl D., Irmler M., Römer I., Mader M.T., Mao L., Zabel C., Angelis M.H., Beckers J., Klose J. (2008). Transcriptome and proteome analysis of early embryonic mouse brain development. Proteomics.

[13] Graveley B.R., Brooks A.N., Carlson J.W., Duff M.O., Landolin J.M., Yang L., Artieri G.G., Baren M.J., Boley N., Booth B.W. (2011). The developmental transcriptome of *Drosophila melanogaster*. Nature.

[14] Liu Z., Liu S., Yao J., Bao L., Zhang J., Li Y., Jiang C., Sun L., Wang R., Zhang Y. (2016). The channel catfish genome sequence provides insights into the evolution of scale formation in teleosts. Nat. Commun..

[15] Martin J.A., Wang Z. (2011). Next-generation transcriptome assembly. Nat. Rev. Genet..

[16] Wang Z., Gerstein M., Snyder M. (2009). RNA-Seq: A revolutionary tool for transcriptomics. Nat. Rev. Genet..

[17] Langfelder P., Horvath S. (2008). WGCNA: An R package for weighted correlation network analysis. BMC Bioinform..

[18] Deng S.P., Zhu L., Huang D.S. (2015). Mining the bladder cancer-associated genes by an integrated strategy for the construction and analysis of differential co-expression networks. BMC Genom..

[19] Miller J.A., Horvath S., Geschwind D.H. (2010). Divergence of human and mouse brain transcriptome highlights Alzheimer disease pathways. Proc. Natl. Acad. Sci. USA.

[20] Xue Z., Huang K., Cai C., Cai L., Jiang C.-Y., Feng Y., Liu Z., Zeng Q., Cheng L., Sun Y.E. (2013). Genetic programs in human and mouse early embryos revealed by single-cell RNA sequencing. Nature.

[21] Clarke C., Madden S.F., Doolan P., Aherne S.T., Joyce H., O’Driscoll L., Gallagher W.M., Hennessy B.T., Moriarty M., Crown J. (2013). Correlating transcriptional networks to breast cancer survival: A large-scale coexpression analysis. Carcinogenesis.

[22] Zhang B., Horvath S. (2005). A general framework for weighted gene co-expression network analysis. Stat. Appl. Genet. Mol. Biol..

[23] Esposti D.D., Almunia C., Guery M.A., Koenig N., Armengaud J., Chaumot A., Geffard O. (2019). Co-expression network analysis identifies gonad-and embryo-associated protein modules in the sentinel species Gammarus fossarum. Sci. Rep..

[24] Zhang J., Nie Q., Si C., Wang C., Chen Y., Sun W., Pan L., Guo J., Kong J., Cui Y. (2019). Weighted gene co-expression network analysis for RNA-sequencing data of the varicose veins transcriptome. Front. Physiol..

[25] Zhao W., Langfelder P., Fuller T., Dong J., Li A., Hovarth S. (2010). Weighted gene coexpression network analysis: State of the art. J. Biopharm. Stat..

[26] Kadarmideen H.N., Watson-Haigh N.S. (2012). Building gene co-expression networks using transcriptomics data for systems biology investigations: Comparison of methods using microarray data. Bioinformation.

[27] Makeeva A., Emel’yanova N. (1993). Early development of the channel catfish, Ictalurus punctatus. J. Lchthyol..

[28] Herpin A., Lelong C., Favrel P. (2004). Transforming growth factor-β-related proteins: An ancestral and widespread superfamily of cytokines in metazoans. Dev. Comp. Immunol..

[29] Safran M., Dalah I., Alexander J., Rosen N., Stein T.I., Shmoish M., Nativ N., Bahir I., Doniger T., Krug H. (2010). GeneCards Version 3: The human gene integrator. Database.

[30] Belinky F., Nativ N., Stelzer G., Zimmerman S., Stein T.I., Safran M., Lancet D. (2015). PathCards: Multi-source consolidation of human biological pathways. Database.

[31] Hino J., Matsuo H., Kangawa K. (1999). Bone morphogenetic protein-3b (BMP-3b) gene expression is correlated with differentiation in rat calvarial osteoblasts. Biochem. Biophys. Res. Commun..

[32] Matsumoto Y., Otsuka F., Hino J., Miyoshi T., Takano M., Miyazato M., Kangawa K. (2012). Bone morphogenetic protein-3b (BMP-3b) inhibits osteoblast differentiation via Smad2/3 pathway by counteracting Smad1/5/8 signaling. Mol. Cell. Endocrinol..

[33] Angioni M., Denotti A., Pinna S., Sanna C., Montisci F., Dessole G., Cauli A. (2019). Spa therapy induces clinical improvement and protein changes in patients with chronic back pain. Reumatismo.

[34] Lavon N., Yanuka O., Benvenisty N. (2006). The effect of overexpression of Pdx1 and Foxa2 on the differentiation of human embryonic stem cells into pancreatic cells. Stem Cells.

[35] Wederell E.D., Bilenky M., Cullum R., Thiessen N., Dagpinar M., Delaney A., Varhol R., Zhao Y., Zeng T., Bernier B. (2008). Global analysis of in vivo Foxa2-binding sites in mouse adult liver using massively parallel sequencing. Nucleic Acids Res..

[36] Jeong J.W., Kwak I., Lee K.Y., Kim T.H., Large M.J., Stewart C.L., Kaestner K.H., Lydon J.P., DeMayo F.J. (2010). Foxa2 is essential for mouse endometrial gland development and fertility. Biol. Reprod..

[37] Lee C.S., Sund N.J., Vatamaniuk M.Z., Matschinsky F.M., Stoffers D.A., Kaestner K.H. (2002). Foxa2 controls Pdx1 gene expression in pancreatic β-cells in vivo. Diabetes.

[38] Wan H., Dingle S., Xu Y., Besnard V., Kaestner K.H., Ang S.-L., Wert S., Stahiman M.T., Whitsett J.A. (2005). Compensatory roles of Foxa1 and Foxa2 during lung morphogenesis. J. Biol. Chem..

[39] Kawaguchi M., Yasumasu S., Hiroi J., Naruse K., Inoue M., Iuchi I. (2006). Evolution of teleostean hatching enzyme genes and their paralogous genes. Dev. Genes. Evol..

[40] Hishida R., Ishihara T., Kondo K., Katsura I. (1996). hch-1, a gene required for normal hatching and normal migration of a neuroblast in C. elegans, encodes a protein related to TOLLOID and BMP-1. EMBO J..

[41] Rawlings N.D., Barrett A.J. (1995). Evolutionary families of metallopeptidases. Meth. Enzymol..

[42] Schramm S., Fraune J., Naumann R., Hernandez-Hernandez A., Höög C., Cooke H.J., Alsheimer M., Benavente R. (2011). A novel mouse synaptonemal complex protein is essential for loading of central element proteins, recombination, and fertility. PLoS Genet..

[43] UniProt Consortium (2018). UniProt: A worldwide hub of protein knowledge. Nucleic Acids Res..

[44] Ruzicka L., Howe D.G., Ramachandran S., Toro S., Van Slyke C.E., Bradford Y.M., Eagle A., Fashena D., Frazer K., Kalita P. (2019). The Zebrafish Information Network: New support for non-coding genes, richer Gene Ontology annotations and the Alliance of Genome Resources. Nucleic Acids Res..

[45] Figeac N., Mohamed A.D., Sun C., Schönfelder M., Matallanas D., Garcia-Munoz A., Missiaglia E., Collie-Duguid E., Mello V.D., Pobbati A.V. (2019). Vgll3 operates via Tead1, Tead3 and Tead4 to influence myogenesis in skeletal muscle. J. Cell Biol..

[46] Kjærner-Semb E., Ayllon F., Kleppe L., Sørhus E., Skaftnesmo K., Furmanek T., Segafredo F.T., Thorsen A., Fjelldal P.G., Hansen T. (2018). Vgll3 and the Hippo pathway are regulated in Sertoli cells upon entry and during puberty in Atlantic salmon testis. Sci. Rep..

[47] Shima Y., Copeland N.G., Gilbert D.J., Jenkins N.A., Chisaka O., Takeichi M., Uemura T. (2002). Differential expression of the seven-pass transmembrane cadherin genes Celsr1-3 and distribution of the Celsr2 protein during mouse development. Dev. Dyn..

[48] Wada H., Tanaka H., Nakayama S., Iwasaki M., Okamoto H. (2006). Frizzled3a and Celsr2 function in the neuroepithelium to regulate migration of facial motor neurons in the developing zebrafish hindbrain. Development.

[49] Poynter S., Monjo A., DeWitte-Orr S. (2018). Identification of three class A scavenger receptors from rainbow trout (*Oncorhynchus mykiss*): SCARA3, SCARA4, and SCARA5. Fish Shellfish Immunol..

[50] Wilson P.M., Fryer R.H., Fang Y., Hatten M.E. (2010). Astn2, a novel member of the astrotactin gene family, regulates the trafficking of ASTN1 during glial-guided neuronal migration. J. Neurosci..

[51] Ni T., Harlos K., Gilbert R. (2016). Structure of astrotactin-2: A conserved vertebrate-specific and perforin-like membrane protein involved in neuronal development. Open Biol..

[52] Sassa T., Aizawa H., Okamoto H. (2007). Visualization of two distinct classes of neurons by *gad2* and *zic1* promoter/enhancer elements in the dorsal hindbrain of developing zebrafish reveals neuronal connectivity related to the auditory and lateral line systems. Dev. Dyn..

[53] Kumari S., Mayor S. (2008). ARF1 is directly involved in dynamin-independent endocytosis. Nat. Cell Biol..

[54] Feng Y., Walsh C.A. (2004). Mitotic spindle regulation by *Nde1* controls cerebral cortical size. Neuron.

[55] Alkuraya F.S., Cai X., Emery C., Mochida G.H., Al-Dosari M.S., Felie J.M., Hill R.S., Barry B.J., Partlow J.N., Gascon G.G. (2011). Human mutations in NDE1 cause extreme microcephaly with lissencephaly. Am. J. Hum. Genet..

[56] Pertz O., Hodgson L., Klemke R.L., Hahn K.M. (2006). Spatiotemporal dynamics of RhoA activity in migrating cells. Nature.

[57] Eide E.J., Virshup D.M. (2001). Casein kinase I: Another cog in the circadian clockworks. Chronobiol. Int..

[58] Schittek B., Sinnberg T. (2014). Biological functions of casein kinase 1 isoforms and putative roles in tumorigenesis. Mol. Cancer.

[59] Pongracz J.E., Stockley R.A. (2006). Wnt signalling in lung development and diseases. Respir. Res..

[60] Patapoutian A., Reichardt L.F. (2000). Roles of Wnt proteins in neural development and maintenance. Curr. Opin. Neurol..

[61] Klaus A., Birchmeier W. (2008). Wnt signalling and its impact on development and cancer. Nat. Rev. Cancer.

[62] Kim K.R., Park S.H., Kim H.S., Rhee K.H., Kim B.G., Kim D.G., Part M.S., Kim H.J., Kim S., Han B.W. (2013). Crystal structure of human cytosolic aspartyl-tRNA synthetase, a component of multi-tRNA synthetase complex. Proteins.

[63] Fröhlich D., Suchowerska A.K., Spencer Z.H., Jonquieres G.V., Klugmann C.B., Bongers A., Delerue F., Stefen H., Lttner L.M., Fath T. (2017). In vivo characterization of the aspartyl-tRNA synthetase DARS: Homing in on the leukodystrophy HBSL. Neurobiol. Dis..

[64] Diodato D., Ghezzi D., Tiranti V. (2014). The mitochondrial aminoacyl tRNA synthetases: Genes and syndromes. Int. J. Cell Biol..

[65] Taft R.J., Vanderver A., Leventer R.J., Damiani S.A., Simons C., Grimmond S.M., Miller D., Schmidt J., Lockhart P.J., Pope K. (2013). Mutations in DARS cause hypomyelination with brain stem and spinal cord involvement and leg spasticity. Am. J. Hum. Genet..

[66] Kikuchi J., Furukawa Y., Kubo N., Tokura A., Hayashi N., Nakamura M., Matsuda M., Sakurabayashi I. (2000). Induction of ubiquitin-conjugating enzyme by aggregated low density lipoprotein in human macrophages and its implications for atherosclerosis. Arterioscler. Thromb. Vasc. Biol..

[67] David Y., Ziv T., Admon A., Navon A. (2010). The E2 ubiquitin-conjugating enzymes direct polyubiquitination to preferred lysines. J. Biol. Chem..

[68] Kanerva K., Mäkitie L.T., Pelander A., Heiskala M., Andersson L.C. (2008). Human ornithine decarboxylase paralogue (ODCp) is an antizyme inhibitor but not an arginine decarboxylase. Biochem. Eng. J..

[69] Hallen L., Burki Y., Ebeling M., Broger C., Siegrist F., Oroszlan-Szovik K., Bohrmann B., Certa U., Foser S. (2007). Antiproliferative activity of the human IFN-α-inducible protein IFI44. J. Interferon Cytokine Res..

[70] Haralambieva I.H., Ovsyannikova I.G., Kennedy R.B., Larrabee B.R., Zimmermann M.T., Grill D.E., Schaid D.J., Poland G.A. (2017). Genome-wide associations of CD46 and IFI44L genetic variants with neutralizing antibody response to measles vaccine. Hum. Genet..

[71] Kong J., Li L., Zhimin L., Yan J., Ji D., Chen Y., Wu Y., Xu C., Shao H., Wang J. (2009). Potential protein biomarkers for systemic lupus erythematosus determined by bioinformatics analysis. Comput. Biol. Chem..

[72] Levy M., Elkoshi N., Barber-Zucker S., Hoch E., Zarivach R., Hershfinkel M., Sekler I. (2019). Zinc transporter 10 (ZnT10)-dependent extrusion of cellular Mn^2+^ is driven by an active Ca^2+^-coupled exchange. J. Biol. Chem..

[73] Hull M.L., Johan M.Z., Hodge W.L., Robertson S.A., Ingman W.V. (2012). Host-derived TGFB1 deficiency suppresses lesion development in a mouse model of endometriosis. Am. J. Pathol..

[74] Ingman W.V., Robertson S.A. (2009). The essential roles of TGFB1 in reproduction. Cytokine Growth Factor Rev..

[75] Manos P., Bryan G.K., Edmond J. (1991). Creatine kinase activity in postnatal rat brain development and in cultured neurons, astrocytes, and oligodendrocytes. J. Neurochem..

[76] Ventura-Clapier R., Mekhfi H., Vassort G. (1987). Role of creatine kinase in force development in chemically skinned rat cardiac muscle. J. Gen. Physiol..

[77] Chauvigné F., Zapater C., Cerdà J. (2011). Role of aquaporins during teleost gametogenesis and early embryogenesis. Front. Physiol..

[78] Sumanas S., Jorniak T., Lin S. (2005). Identification of novel vascular endothelial–specific genes by the microarray analysis of the zebrafish cloche mutants. Blood.

[79] Cannon J., Place E., Eve A., Bradshaw C., Sesay A., Morrell N., Smith J. (2013). Global analysis of the haematopoietic and endothelial transcriptome during zebrafish development. Mech. Dev..

[80] Sedykh I., Yoon B., Roberson L., Moskvin O., Dewey C.N., Grinblat Y. (2017). Zebrafish zic2 controls formation of periocular neural crest and choroid fissure morphogenesis. Dev. Biol..

[81] Frank C.L., Liu F., Wijayatunge R., Song L., Biegler M.T., Yang M.G., Vockley C.M., Safi A., Gersbach C.A., Crawford G.E. (2015). Regulation of chromatin accessibility and Zic binding at enhancers in the developing cerebellum. Nat. Neurosci..

[82] Hillegass J.M. (2008). The role of matrix metalloproteinases in zebrafish (Danio rerio) embryogenesis and their regulation by glucocorticoids. Master’s Thesis.

[83] Pociute K., Schumacher J.A., Sumanas S. (2019). *Clec14a* genetically interacts with *Etv2* and *Vegf* signaling during vasculogenesis and angiogenesis in zebrafish. BMC Dev. Biol..

[84] Barcia G., Fleming M.R., Deligniere A., Gazula V.R., Brown M.R., Langouet M., Chen H., Kronengold J., Abhyankar A., Cilio R. (2012). *De novo* gain-of-function KCNT1 channel mutations cause malignant migrating partial seizures of infancy. Nat. Genet..

[85] Oliver K.L., Franceschetti S., Milligan C.J., Muona M., Mandelstam S.A., Canafoglia L., Boguszewska-Chachulska A.M., Korczyn A.D., Bisulli F., Di Bonaventura C. (2017). Myoclonus epilepsy and ataxia due to KCNC1 mutation: Analysis of 20 cases and K^+^ channel properties. Ann. Neurol..

[86] Smitherman R.O., Dunham R.A., Tave D. (1983). Review of catfish breeding research 1969–1981 at Auburn University. Aquaculture.

[87] Backenstose N.A. (2018). Histological Evaluation of the Development of Respiratory Structures in Channel Catfish (*Ictalurus punctatus*) and Tra (*Pangasianodon hypophthalmus*). Master’s Thesis.

[88] Caporaso J.G., Lauber C.L., Walters W.A., Berg-Lyons D., Huntley J., Fierer N., Owens S.M., Betley J., Fraser L., Bauer M. (2012). Ultra-high-throughput microbial community analysis on the Illumina HiSeq and MiSeq platforms. ISME J..

[89] Wingett S.W., Andrews S. (2018). FastQ Screen: A tool for multi-genome mapping and quality control. F1000Research.

[90] Bolger A.M., Lohse M., Usadel B. (2014). Trimmomatic: A flexible trimmer for Illumina sequence data. Bioinformatics.

[91] Dobin A., Davis C.A., Schlesinger F., Drenkow J., Zaleski C., Jha S., Batut P., Chaisson M., Gingeras T.R. (2013). STAR: Ultrafast universal RNA-seq aligner. Bioinformatics.

[92] Anders S., Pyl P.T., Huber W. (2015). HTSeq—A Python framework to work with high-throughput sequencing data. Bioinformatics.

[93] Love M.I., Huber W., Anders S. (2014). Moderated estimation of fold change and dispersion for RNA-seq data with DESeq2. Genome Biol..

[94] Yu G., Wang L.G., Han Y., He Q.Y. (2012). clusterProfiler: An R package for comparing biological themes among gene clusters. OMICS.

[95] Smoot M.E., Ono K., Ruscheinski J., Wang P.L., Ideker T. (2010). Cytoscape 2.8: New features for data integration and network visualization. Bioinformatics.

