# Supplementary material for: Deep Transcriptomic Analysis Reveals the Dynamic Developmental Progression during Early Development of Channel Catfish (Ictalurus punctatus)
Source: Int J Mol Sci. 2020 Aug 2;21(15):5535. doi: 10.3390/ijms21155535 (PMC7432863; doi:10.3390/ijms21155535)
Supplement: Supplementary file 1 [file ijms-21-05535-s001.zip › FigureS1-S2.pdf]

Figure S1. Overview of RNA-Seq mapping in channel catfish (*Ictalurus punctatus*).

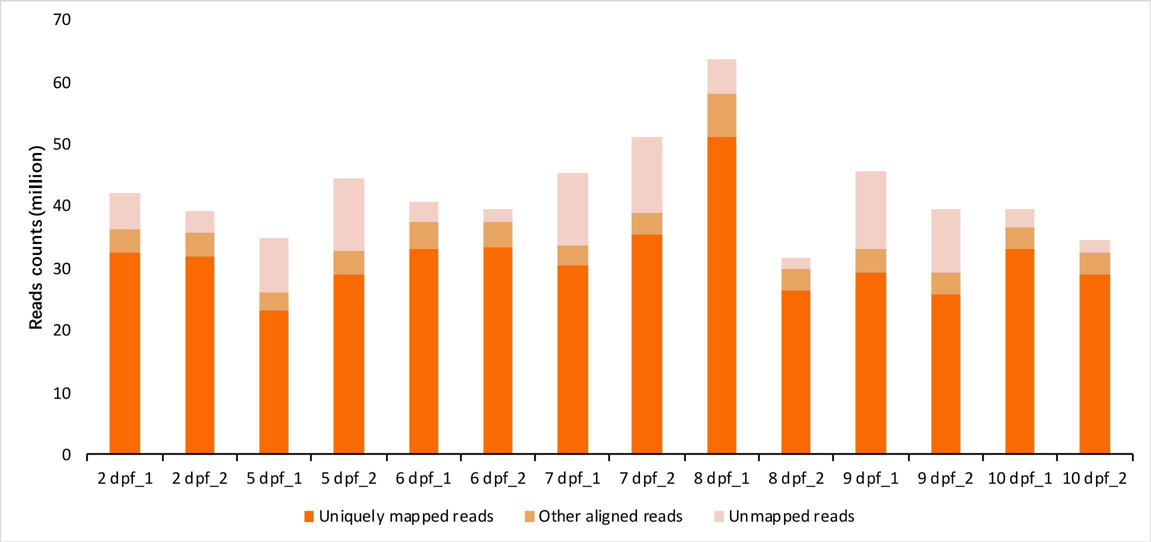

Figure S2. Analysis of network topology for different soft thresholding powers in channel catfish (*Ictalurus punctatus*). In the left diagram, y-axis is the scale-free fit index as a function of the soft-thresholding power (x-axis). The right diagram shows the mean connectivity (degree, y-axis) as a function of the soft-thresholding power (x-axis).

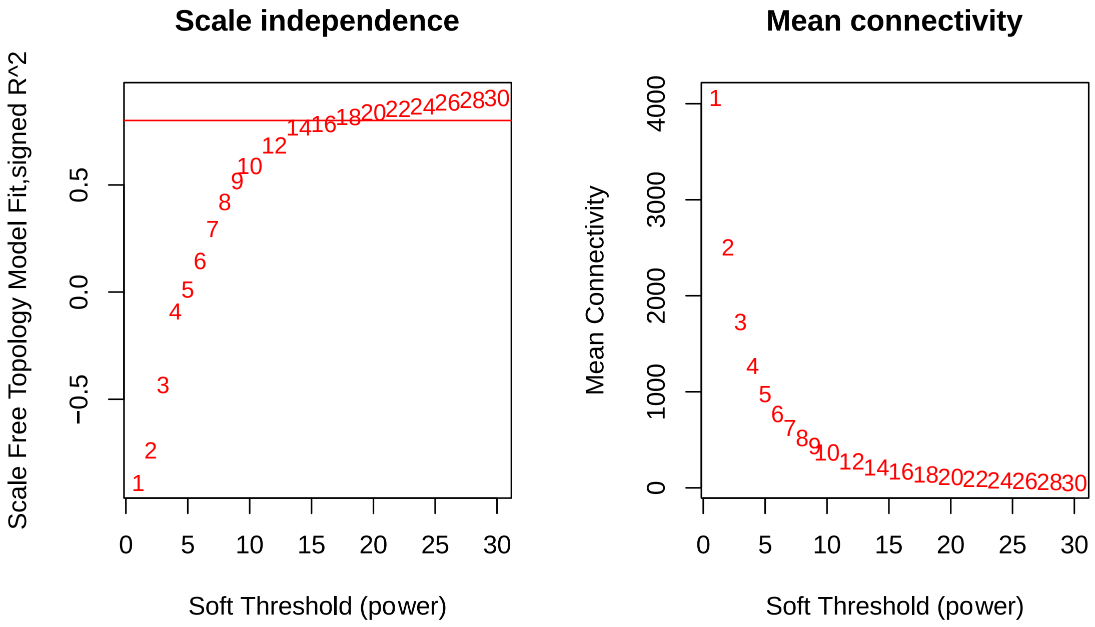

~~**Table S1.** Summary statistics of the transcriptome sequencing in channel catfish (*Ictalurus punctatus*) early development stages (CCN1-N2; channel catfish days post fertilization (dpf)- replicate number).~~

Sampling	No. of raw reads	No. of clean reads	%GC	No. of mapped reads	Mapping rate (%)
CC2-1	91,669,324	42,131,732	49	36,303,934	86.16%
CC2-2	89,890,166	39,249,396	50	35,763,124	91.12%
CC5-1	64,585,468	34,709,236	48	261,03,146	75.21%
CC5-2	84,453,314	44,511,770	48	32,883,240	73.87%
CC6-1	100,421,168	40,793,066	50	37,564,304	92.08%
CC6-2	94,323,974	39,364,512	48	37,419,614	95.06%
CC7-1	87,494,136	45,224,692	47	33,714,328	74.55%

~~CC7-2~~	~~98,318,154~~	~~51,048,372~~	~~47~~	~~39,055,300~~	~~76.51%~~
~~CC8-1~~	~~116,489,572~~	~~63,382,006~~	~~50~~	~~58,254,748~~	~~91.91%~~
~~CC8-2~~	~~73,963,212~~	~~31,525,758~~	~~49~~	~~29,772,108~~	~~94.44%~~
~~CC9-1~~	~~92,613,334~~	~~45,676,882~~	~~47~~	~~33,003,294~~	~~72.26%~~
~~CC9-2~~	~~79,448,748~~	~~39,374,808~~	~~48~~	~~29,202,904~~	~~74.17%~~
~~CC10-1~~	~~89,215,722~~	~~39,707,608~~	~~49~~	~~36,730,700~~	~~92.5%~~
~~CC10-2~~	~~95,788,206~~	~~34,652,038~~	~~48~~	~~32,555,886~~	~~93.95%~~
